# Baryon chiral perturbation theory extended beyond the low-energy region

**DOI:** 10.1140/epjc/s10052-015-3728-7

**Published:** 2015-10-22

**Authors:** E. Epelbaum, J. Gegelia, Ulf-G. Meißner, De-Liang Yao

**Affiliations:** Institut für Theoretische Physik II, Ruhr-Universität Bochum, 44780 Bochum, Germany; Institute for Advanced Simulation, Institut für Kernphysik and Jülich Center for Hadron Physics, Forschungszentrum Jülich, 52425 Jülich, Germany; Tbilisi State University, 0186 Tbilisi, Georgia; Helmholtz Institut für Strahlen- und Kernphysik and Bethe Center for Theoretical Physics, Universität Bonn, 53115 Bonn, Germany

## Abstract

We consider an extension of the one-nucleon sector of baryon chiral perturbation theory beyond the low-energy region. The applicability of this approach for higher energies is restricted to small scattering angles, i.e. the kinematical region, where the quark structure of hadrons cannot be resolved. The main idea is to re-arrange the low-energy effective Lagrangian according to a new power counting and to exploit the freedom of the choice of the renormalization condition for loop diagrams. We generalize the extended on-mass-shell scheme for the one-nucleon sector of baryon chiral perturbation theory by choosing a sliding scale, that is, we expand the physical amplitudes around kinematical points beyond the threshold. This requires the introduction of complex-valued renormalized coupling constants, which can be either extracted from experimental data, or calculated using the renormalization group evolution of coupling constants fixed in threshold region.

## Introduction

Effective field theories (EFTs) of the strong interaction started with the pioneering work by Weinberg [1]. The main idea of this approach is that by considering the most general effective Lagrangian of dynamical fields corresponding to the relevant light degrees of freedom, which is invariant under all symmetries of quantum chromodynamics (QCD), one can reproduce the non-trivial low-energy structure of the *S*-matrix of QCD. Contributions of heavy degrees of freedom are analytic at low energies and therefore can be represented by a systematic expansion of the effective Lagrangian in powers of quark masses and derivatives acting on fields. The Goldstone-boson sector of chiral EFT, called chiral perturbation theory (ChPT), has been worked out in detail in Ref. [2]. The inclusion of nucleons in this framework proved to be more complicated due to the non-vanishing chiral limit value of the nucleon mass [3]. The encountered non-trivial problem of power counting in manifestly Lorentz invariant formulations of baryon ChPT (BChPT) has first been resolved by applying the heavy baryon approach [4–7]. Later it has been suggested that the power counting can also be respected within the original manifestly Lorentz invariant formulation of BChPT by applying an appropriate renormalization scheme [8–13]. The solution of the power counting problem in manifestly Lorentz invariant formulations of BChPT is based on the observation that the power counting violating parts of loop diagrams are polynomial in quark masses and external momenta and can be subtracted systematically by renormalizing the parameters of the effective Lagrangian. A detailed discussion of conceptual issues and applications of BChPT to various processes can be found, e.g., in Refs. [14, 15].

In the current work we extend the applicability of BChPT beyond the low-energy region under the condition that the scattering angles are small.

Below we demonstrate the main idea behind the extension of applicability of BChPT on an example of a Taylor expansion of a function of one variable. We treat the function the way we do for the *tree-order* scattering amplitudes generated by a chirally invariant effective Lagrangian. Let us consider a function *f*(*x*) which is analytic at $x=0$. It can be expanded in a convergent Taylor series for small *x*

1 $$\begin{aligned} f(x)=x^i (a_0+a_1 x+ a_2 x^2 + \cdots ), \end{aligned}$$

where $a_j$ are numerical coefficients and *i* is either zero, or some integer number (in the case of BChPT the analog of *i* takes different values depending on the considered physical amplitudes). For sufficiently small *x* the function *f*(*x*) can be well approximated by the first few terms of the series of Eq. (1). For larger *x* we may expand around another point, say $x_0$. If the function *f*(*x*) contains a singularity close to $x_0$ (this is the case for BChPT due to the presence of resonances), we isolate the singular part and expand the remaining non-singular piece

2 $$\begin{aligned} f(x)&= x^i \phi (x)=x^i\left[ \phi _\mathrm{reg}(x)+\phi _\mathrm{sing}(x)\right] \nonumber \\&=x^i \phi _\mathrm{sing}(x) + x^i \bigg [\phi _\mathrm{reg}(x_0)+\phi _\mathrm{reg}'(x_0) (x-x_0)\nonumber \\&\quad + \frac{\phi _\mathrm{reg}''(x_0)}{2!} (x-x_0)^2 + \cdots \bigg ]. \end{aligned}$$

Let us emphasize that while usually one would use the standard Taylor series expansion around $x=x_0$ for the regular part of the function *f*(*x*) (i.e. would expand $x^i \phi _\mathrm{reg}(x)$ not just $\phi _\mathrm{reg}$) the alternative expansion given by Eq. (2) also provides a consistent convergent series and, as will be seen later, an analogous expansion is well suited for physical amplitudes generated by the chirally invariant Lagrangian. In particular, if one cuts the series in Eq. (2) at any finite order of $(x-x_0)^N$, the obtained result will be an $i+N$th order polynomial in *x*, i.e. it has the same structure as the $i+N$th order result of the series of Eq. (1), however, with different coefficients of the polynomial. This almost trivial feature is important for BChPT as it guarantees that the effective Lagrangian for higher energies at any finite order is obtained by re-arranging a finite number of chirally invariant terms in the standard effective Lagrangian designed for the expansion at low-energies.

We extend the applicability of BChPT beyond the low-energy region by re-arranging the chirally invariant terms of the standard low-energy effective Lagrangian and by introducing a generalization of the extended on-mass-shell (EOMS) scheme of Refs. [11–13]. We obtain an EFT with new well-defined power counting rules. Loop diagrams contributing to physical amplitudes violate this power counting. However, the divergent parts as well as power counting violating pieces can be subtracted by applying a generalization of the EOMS scheme. The subtracted terms are absorbed in the redefinition of parameters of the re-arranged effective Lagrangian. As the subtractions are made above the threshold, the corresponding counter terms contain imaginary parts. This means that the renormalized parameters become complex. Thus, the suggested modification of the EOMS scheme belongs to the class of complex mass schemes (CMS) first considered in Refs. [16, 17]. One might be concerned about unitarity within the CMS because of the use of complex renormalized parameters, however, this issue has been discussed in detail recently in Ref. [18] (see also Ref. [19]).

Considering physical amplitudes of the one-nucleon sector within the new approach, we obtain a finite number of diagrams at any finite order, i.e. the calculations are perturbative. It is not surprising that this framework, which uses the hadronic degrees of freedom for higher energies, can be applied only close to the forward direction, where the quark structure of hadrons cannot be resolved. Analogously to the standard low-energy EFT, the radius of convergence of perturbative series is determined by the nearest non-analytic structure. The branch points and cuts of the *S*-matrix of QCD are generated by loop diagrams in the EFT framework. On the other hand, poles represent non-perturbative effects. Therefore, the appearance of poles in the *S*-matrix requires the inclusion of the corresponding fields as explicit degrees of freedom in the effective Lagrangian or performing some kinds of non-perturbative resummations. That is, all resonances which appear at the considered energies must be included as dynamical degrees of freedom in the effective Lagrangian within our new perturbative framework.

Our paper is organized as follows. In Sect. 2 we consider pion–nucleon scattering at tree order beyond the threshold region and the corresponding re-arrangement of the chirally symmetric effective Lagrangian. While our new approach is applicable to the one-nucleon sector of BChPT in general, in the current work pion–nucleon scattering is considered in some detail as a demonstration of the method. Section 3 addresses the issue of renormalization introducing the EOMS scheme with a sliding scale. The scale-dependence of renormalized coupling constants and the phase shifts of the pion–nucleon scattering in the threshold region applying the EOMS scheme with the sliding scale are considered in Sects. 4 and 5 contains conclusions. In the appendix we give some explicit expressions and briefly touch upon the issue of complex renormalized parameters and unitarity.

## Pion–nucleon scattering at tree order and the effective Lagrangian of BChPT

We consider the process $\pi ^a(q) N(p)\rightarrow \pi ^{a'}(q') N(p')$ assuming exact isospin-symmetry. Here, *a* and $a'$ are Cartesian isospin indices. The Mandelstam variables are defined in standard form as $s=(p+q)^2=(p'+q')^2$, $t=(q-q')^2=(p-p')^2$, and $u=(p-q')^2=(p'-q)^2$. They fulfill the identity $s+t+u=2 m_N^2+2 M_\pi ^2$, where $m_N$ and $M_\pi$ are the physical masses of the nucleon and the pion, respectively. We parameterize the pion–nucleon scattering amplitude in the standard way [22]:3 In BChPT it is convenient to utilize the *D* and *B* amplitudes as functions of *t* and $\nu$, where $\nu =(s-u)/(4 m_N)$. Due to the crossing symmetry the amplitudes $X\in \{D^+,\, D^-/\nu ,\,B^+/\nu ,\, B^-\}$ are even functions of $\nu$. It is useful to consider the difference between the full pion–nucleon scattering amplitude and the pseudovector Born term expanded around the point $\nu =t=0$ [22, 23] (subthreshold expansion)

4 $$\begin{aligned} X(\nu ,t) = X_{pv}(\nu ,t) + \sum _{i,j=0}^\infty x_{ij} \nu ^{2\,i} t^j, \end{aligned}$$

where $X_{pv}(\nu ,t)$ are the pseudovector Born terms and $x\in \{d^+,\, d^-,\,b^+,\, b^-\}$.

Spontaneously broken chiral symmetry predicts that $d_{00}^+=0$ and $d_{00}^-=1/(2 F^2)$ in the chiral limit of vanishing up and down quark masses, where *F* is the pion decay constant in that limit. Taking into account this observation, the one-particle irreducible tree-order contributions of the effective Lagrangian can be parameterized as

5 $$\begin{aligned} \begin{aligned} D^+&= d_0^+(t,M)+d_2^+(t,M) \nu ^2 + d_4^+(t,M) \nu ^4 + \cdots ,\\ D^-&= d_1^-(t,M) \nu + d_3^-(t,M) \nu ^3 + \cdots ,\\ B^+&= b_1^+(t,M)\nu + b_3^+ (t,M)\nu ^3 + \cdots ,\\ B^-&= b_0^-(t,M) + b_2^-(t,M) \nu ^2 + \cdots , \end{aligned} \end{aligned}$$

where $d_j^{\pm }(t,M)$ and $b_j^{\pm }(t,M)$ are Taylor series in *t* and *M*, with *M* the leading-order term in the chiral expansion of the pion mass. The coefficients of series in Eq. (5) also contain chiral logarithms (i.e. terms $\sim \ln M$, which are *not* contained in the effective Lagrangian but rather generated by the on-shell condition of external pions). In the low-energy region various contributions to the amplitudes are organized according to the power counting which assigns order $q^2$ to *t*, $q^1$ to $\nu$ and order $q^1$ to *M*, with *q* denoting a small quantity. The amplitudes of a given order in Eq. (5) are generated by terms of the low-energy effective Lagrangian of corresponding orders. Terms of the effective Lagrangian generating (leading) tree diagram contributions of order $q^N$ count as order *N*.

To consider the tree-order amplitudes beyond the threshold region we re-expand them at $\nu ^2=\mu ^2$ as follows:

6 $$\begin{aligned} D^+= & {} d_0^+(t,M)+\nu ^2 \left[ \tilde{d}_2^+(t,M) + \tilde{d}_4^+(t,M) (\nu ^2-\mu ^2) + \cdots \right] ,\nonumber \\ D^-= & {} d_1^-(t,M) \nu + \nu ^3 \left[ \tilde{d}_3^-(t,M) + \tilde{d}_5^-(t,M) (\nu ^2-\mu ^2) + \cdots \right] ,\nonumber \\ B^+= & {} \nu \left[ \tilde{b}_1^+(t,M) + b_3^+(t,M) (\nu ^2-\mu ^2) + \cdots \right] ,\nonumber \\ B^-= & {} \tilde{b}_0^-(t,M) + \tilde{b}_2^-(t,M) (\nu ^2-\mu ^2) + \cdots . \end{aligned}$$

Note here that the different treatment of $D^+$ and $B^{-}$ is caused by the fact that $d_{00}^+=0$, i.e. we keep at each order of the new exapnsion the property that $D^+ =0$ for $t=M=\nu =0$. Analogously, the fixed value $d_{00}^-=1/(2 F^2)$ causes the different treatment of $D^{-}$ and $B^{+}$. The power series expansion of Eq. (6) can be generated by an effective Lagrangian with the same structures as contained in the standard effective Lagrangian constructed for the near-threshold region, however, the terms have to be re-arranged according to new power counting rules. In particular, considering now *Q* as a small parameter, *t* counts as order $Q^2$, while *M* and $\nu ^2-\mu ^2$ count as order $Q^1$. It is understood that in the Taylor series of $\tilde{d}_i^{\pm }(t,M)$ and $\tilde{b}_i^{\pm }(t,M)$ a finite number of terms, corresponding to the given specified order of accuracy, are retained. Terms of the effective Lagrangian, i.e. combinations of the chirally invariant structures, which generate contributions of order $Q^N$ at tree level count as order $Q^N$. The re-arranged effective Lagrangian is organized as an expansion according to these orders. At any finite order it contains a finite number of chirally invariant structures, terms which coincide with those of the standard Lagrangian, however, the assigned orders are different and the coupling constants are also different. In particular, each chirally invariant term of the original low-energy effective Lagrangian with a given low-energy coupling constant is split into an infinite number of contributions in an infinite number of terms of the re-arranged Lagrangian. The sum of coefficients of all these infinite number of contributions of the same chirally invariant structure (in terms of growing orders of the re-arranged effective Lagrangian) reproduces the coefficient of the corresponding term in the standard low-energy effective Lagrangian, at least formally. Notice that if one is comparing the low-energy effective Lagrangian without resonances (as explicit degrees of freedom) to the re-arranged effective Lagrangian with resonances, then one needs to take into account that low-energy coupling constants also get contributions from resonances when they are integrated out.

To be more specific, the lowest-order terms in $D^+$ are proportional to $\nu ^2$, $M^2$ or *t*, which are of order $Q^0$, $Q^2$, and $Q^2$, respectively. Therefore, terms of the low-energy effective Lagrangian of order $q^{2+2 i}$, which give contributions to $D^+$ proportional to $\nu ^{2+2 i}$, count as order $Q^i$. Terms of order $q^{2 i+2 j+2 k}$ giving contributions proportional to $\nu ^{2 i} (M^2)^{j} t^{k}$ ($j+k\ne 0$) count as order $Q^{i+2j+2k}$. Here and below by the order of a given structure is meant the lowest order, to which it contributes.

The leading-order term in $D^-$ is generated by the covariant derivative part of the standard leading-order low-energy pion–nucleon Lagrangian $\mathcal{L}_{\pi N}^{(1)}$ [3], which cannot be re-arranged because it generates the undressed propagator of the nucleon. The first subleading terms are proportional to $\nu ^3$, $\nu M^2$, and $\nu t$, which are of order $Q^0$, $Q^2$, and $Q^2$, respectively. Therefore, terms of the low-energy effective Lagrangian of order $q^{3+2 i}$, which give contributions to $D^-$ proportional to $\nu ^{3+2 i}$, count as order $Q^i$. Terms of order $q^{1+2 i+2 j+2 k}$ giving contributions proportional to $\nu ^{1+2 i} (M^2)^{j} t^{k}$ ($j+k\ne 0$) count as order $Q^{i+2 j+2 k}$.

The lowest-order terms in $B^+$ are proportional to $\nu$, which is of order $Q^0$. Therefore, terms of the low-energy effective Lagrangian of order $q^{3+2 i}$, which give contributions to $B^+$ proportional to $\nu ^{1+2 i}$, count as order $Q^{1+i}$. Terms of the order $q^{3+2 i+2 j+2 k}$ giving contributions proportional to $\nu ^{1+2 i} (M^2)^{j} t^{k}$ ($j+k\ne 0$) count as order $Q^{1+i+2 j+2 k}$. Note here that the amplitudes $B^{\pm }$ are multiplied with , which gives two additional orders of the small parameter *q* in low-energy region and one additional order of *Q* in the higher-energy region. We assign $Q^1$ to the factor  according to its contribution to the cross section in the energy region beyond the threshold.

The lowest-order terms in $B^-$ are proportional to $\nu ^0$, which is of order $Q^0$. Therefore, terms of the low-energy effective Lagrangian of order $q^{2+2 i}$, which give contributions to $B^-$ proportional to $\nu ^{2 i}$, count as order $Q^{1+i}$. Terms of order $q^{2+2 i+2 j+2 k}$, giving contributions to $B^-$ proportional to $\nu ^{2 i} (M^2)^{j} t^{k}$ ($j+k\ne 0$) count as order $Q^{1+i+2 j+2 k}$.

Thus to construct the re-arranged Lagrangian of order *N*, using the above power counting for tree-order contributions, we need to examine all structures of the standard BChPT Lagrangian up to (including) order $2(N+1)+1$ and determine their orders for the region beyond the threshold according to their contributions in the tree-order amplitudes. In addition, we need to re-arrange the structures of the standard low-energy effective Lagrangian in such a way that in tree-order amplitudes power series expansions in terms of $\nu ^2-\mu ^2$ appear. We denote the $k\mathrm{th}$ order re-arranged effective Lagrangian by $\tilde{\mathcal{L}}_{\pi N}^{(k)}$.

**Fig. 1:**
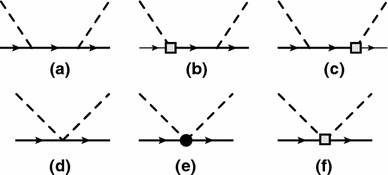
Tree diagrams contributing to the pion–nucleon scattering at $\mathcal{O}(q^3)$. The *solid* and *dashed lines* correspond to the nucleon and the pion, respectively. Crossed diagrams are not shown. Different tree diagrams correspond to different orders

The tree diagrams contributing to the $\pi N$ scattering amplitudes at $q^3$ order are shown in Fig. 1. Below we specify explicitly the amplitudes corresponding to one-particle irreducible tree-order diagrams which are generated by the standard low-energy effective Lagrangian up to including the third order [20, 21]. For the purpose of the re-arranged theory we also include one fourth-order and one fifth-order term:

7 $$\begin{aligned} D^{+}_\mathrm{tree}&= \frac{16\,c_2\,m_N^2\,\nu ^2}{8 F^2 m^2} -\frac{4\,c_1 M^2}{F^2}+\frac{c_3(2 M_\pi ^2-t)}{F^2}\nonumber \\&\quad + \frac{16\,e_{16}\,\nu ^4}{F^2}+\cdots ,\nonumber \\ D^{-}_\mathrm{tree}&= \frac{\nu }{2 F^2}+\frac{4 d_3 \nu ^3}{F^2}\nonumber \\&\quad +\frac{2 \nu \left[ 2 M_\pi ^2(2 d_5+d_1+d_2)-(d_1+d_2) t \right] }{F^2}\nonumber \\&\quad +\frac{f_x \nu ^5}{F^2} + \cdots ,\nonumber \\ B^{+}_\mathrm{tree}&= \frac{4 \left( d_{14}-d_{15}\right) m_N \,\nu }{F^2}+\cdots ,\nonumber \\ B^{-}_\mathrm{tree}&= \frac{1}{2 F^2}+\frac{2 c_4 m_N}{F^2} +\cdots . \end{aligned}$$

Here, *m* and *F* are the nucleon mass and pion decay constant in the chiral limit, respectively, and the $c_i$, $d_i$, and $e_i$ are the low-energy constants of the standard effective Lagrangian [21] and $f_x$ is a linear combination of coupling constants of the fifth-order effective Lagrangian (not yet available in literature).

Below we show the new tree-order expressions obtained by re-arranging these terms. Contributions of different orders are put in square brackets and the corresponding orders of the small parameter *Q* are indicated as subscripts:

8 $$\begin{aligned} D^{+}_\mathrm{tree}= & {} \left[ \frac{16\,\tilde{c}_2\,\nu ^2}{8 F^2}\right] _0 + \left[ \frac{16\,\tilde{e}_{16}\,\nu ^2 (\nu ^2-\mu ^2)}{8 F^2}\right] _1+\cdots ,\nonumber \\ D^{-}_\mathrm{tree}= & {} \left[ \frac{\nu }{2 F^2}+\frac{4 \tilde{d}_3 \nu ^3}{F^2}\right] _0 +\left[ \frac{\tilde{f}_x \nu ^3(\nu ^2-\mu ^2)}{F^2}\right] _1 +\cdots , \nonumber \\ B^{+}_\mathrm{tree}= & {} \left[ \frac{4 \left( \tilde{d}_{14}-\tilde{d}_{15}\right) m \,\nu }{F^2}\right] _0 +\cdots ,\nonumber \\ B^{-}_\mathrm{tree}= & {} \left[ \frac{1}{2 F^2}+\frac{2 \tilde{c}_4 m_N}{F^2}\right] _0+\cdots , \end{aligned}$$

where we kept only zeroth-order terms in the $B^\pm$ amplitudes because of the order $Q^1$ prefactor . The new parameters $\tilde{c}_i$, $\tilde{d}_i$, and $\tilde{e}_i$ depend on $\mu$ and they are related to the original low-energy constants:

9 $$\begin{aligned} c_2 -\Delta c_2= & {} \tilde{c}_2 + \tilde{e}_{16}\mu ^2 +\cdots ,\nonumber \\ c_3 - \Delta c_3= & {} \tilde{c}_3 + 8\,\tilde{e}_{15}\mu ^2 +\cdots ,\nonumber \\ c_4 - \Delta c_4= & {} \tilde{c}_4 + 8\,\tilde{e}_{18}\mu ^2 +\cdots , \end{aligned}$$

where $\Delta c_i$ are the contributions of resonances which need to be included dynamically in an extended effective theory and the ellipses stand for an infinite number of terms with increasing powers of $\mu$.

The leading-order re-arranged effective Lagrangian of the one-nucleon sector generating the leading zeroth-order term in the expansion of Eq. (8) reads

10 $$\begin{aligned} \tilde{\mathcal{L}}_{\pi N}^{(0)}&= \bar{\Psi }\left( i\gamma _\mu D^\mu -m + \frac{1}{2} g_A \gamma _\mu u^\mu \gamma _5 \right) \nonumber \\&\quad \times \Psi -\frac{\tilde{c}_2}{4 m^2}\,\langle u_\mu u_\nu \rangle \,\bar{\Psi }\left( D^\mu D^\nu +h.c. \right) \nonumber \\&\quad \times \Psi + \frac{\tilde{d}_3}{12 m^3}\,\bar{\Psi }\left\{ \left[ u_\mu ,\left[ D_\nu ,u_\lambda \right] \right] \left( D^\mu D^\nu D^\lambda +sym. \right) \right. \nonumber \\&\quad \left. +\,h.c.\right\} \Psi . \end{aligned}$$

Here, $\Psi$ denotes the nucleon field, $D_\mu \Psi = (\partial _\mu +\Gamma _\mu )\Psi$ is the covariant derivative (in the absence of external vector and axial-vector fields) and

11 $$\begin{aligned} u^2=U,\quad u_\mu =iu^{\dagger }\partial _\mu U u^{\dagger },\quad \Gamma _\mu =\frac{1}{2} \,[u^{\dagger },\partial _\mu u], \end{aligned}$$

where *U* is a unimodular unitary $(2\times 2)$ matrix of the Goldstone-boson fields. Terms of the re-arranged effective Lagrangian, corresponding to next-to-leading order contributions explicitly shown in Eq. (8) has the form

12 $$\begin{aligned} \tilde{\mathcal{L}}_{\pi N}^{(1)}&= \frac{\tilde{e}_{16}}{48 m^4} \bigg \{\bar{\Psi }\left[ \langle h_{\lambda \mu } h_{\nu \rho }\rangle D^{\lambda \mu \nu \rho }+h.c.\right] \nonumber \\&\quad \times \Psi +12\,m^2 \mu ^2 \langle u_\mu u_\nu \rangle \bar{\Psi }\left( D^\mu D^\nu +h.c. \right) \Psi \,\bigg \}. \nonumber \\&\quad + i\,\frac{d_{14}-d_{15}}{8 m}\,\bar{\Psi }\bigg \{ \sigma ^{\mu \nu } \bigg \langle \left[ D_\lambda ,u_\mu \right] u_\nu \nonumber \\&\quad - u_\mu \left[ D_\nu ,u_\lambda \right] \bigg \rangle D^\lambda +h.c. \bigg \} \Psi - \frac{\tilde{c}_4}{4} \,\bar{\Psi }\gamma ^\mu \gamma ^\nu \left[ u_\mu ,u_\nu \right] \nonumber \\&\quad \times \Psi + \tilde{f}_x \,\bar{\Psi }\bigg \{ \hat{\mathcal{O}}-\frac{\mu ^2 }{48 m^3} \bigg (\left[ u_\mu ,\left[ D_\nu ,u_\lambda \right] \right] \nonumber \\&\quad \times \left( D^\mu D^\nu D^\lambda +sym. \right) +h.c.\bigg )\bigg \} \Psi , \end{aligned}$$

where by $\hat{\mathcal{O}}$ we indicated a combination of operators of the fifth-order low-energy Lagrangian (not yet available in the literature), which generates the contribution $\sim \nu ^5$ in the $D^-$ amplitude.

## EOMS scheme with sliding scale

**Fig. 2:**
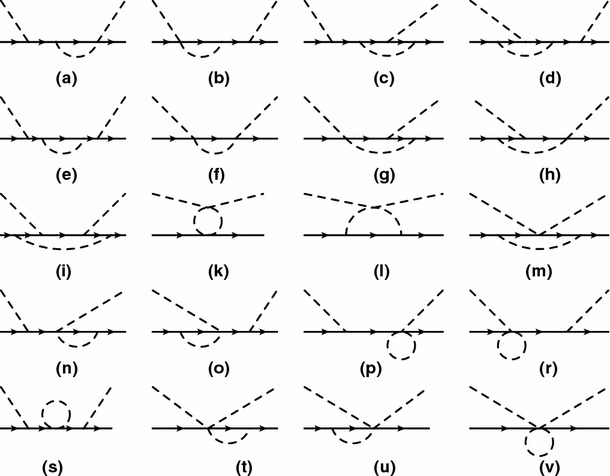
One-loop diagrams contributing to the pion–nucleon scattering at $\mathcal{O}(q^3)$. The solid and dashed lines correspond to the nucleon and the pion, respectively. Crossed diagrams are not shown

To renormalize loop diagrams, we use the EOMS scheme with a sliding scale. In particular, we move the normalization point away from the threshold to larger values of the energy. That is, we take the forward-scattering amplitude at some fixed energy in the chiral limit as an input and calculate the expansion around this point. The renormalized parameters of the effective Lagrangian become complex in this framework. Within this scheme the power counting of the previous section is also applicable to loop diagrams. However, the rules are more complicated for higher-energy regions. In particular, the orders assigned to one-particle irreducible tree diagrams and correspondingly to the effective Lagrangian cannot be directly translated into the rules for loop diagrams. That is, to vertices generated by the re-arranged effective Lagrangian we assign their corresponding orders according to *q*-counting. Next we draw all loop diagrams using these vertices and recalculate the orders of loop diagrams in *q*-counting (low-energy region) to orders of *Q*-counting (high-energy region) analogously to tree-order diagrams. Doing so we assign definite orders of a small parameter *Q* to each loop diagram. Depending on the order of our calculation we identify those diagrams which have to be included. For example, consider diagram f) of Fig. 2. According to the standard power counting it is of order $q^3$. This diagram together with its crossed partner gives a contribution in $D^{-}$ amplitude, proportional to $\nu ^3$. Therefore, recalculating the orders analogously to tree-order diagrams, we find that diagram f) is of order $Q^0$ in higher-energy region.

Loop diagrams do not satisfy the power counting before renormalization is carried out. However, all power counting violating parts are polynomial in external momenta and the pion mass squared and therefore can be canceled (subtracted) by counterterms of the effective Lagrangian. We use the EOMS scheme with a sliding scale as demonstrated below by considering a simple one-loop integral

13 $$\begin{aligned}&B_0(p^2,M^2,m^2) = \frac{(2 \pi )^{4-n}\mu _d^{4-n}}{i\,\pi ^2}\nonumber \\&\quad \times \int \frac{d^nk}{\left[ k^2-M^2+i \delta \right] \left[ (p+k)^2-m^2+i \delta \right] }, \end{aligned}$$

where *n* is the number of space-time dimensions and $\mu _d$ is the scale of dimensional regularization, which should not be confused with our subtraction point. According to standard power counting rules $B_0(p^2,M^2,m^2)$ is of order $q^1$. This power counting can be satisfied by subtracting the integral at $p^2=\mu _p^2$. Note that $\mu _p^2=m^2+2 m \mu$, where $\mu$ is the subtraction point used later in Sect. 4, provided that $p^2$ is identified with the Mandelstam *s* of pion–nucleon scattering. By direct calculation we obtain the following subtraction terms:

14 $$\begin{aligned} B_0^{ST}&= -32\,\pi ^2 \bar{\lambda }-2 \ln \frac{m}{\mu _d}+1+\left( \frac{\mu _p^2}{m^2}-1\right) \nonumber \\&\quad \times \left[ \ln \left( \frac{\mu _p^2}{m^2}-1\right) -i\pi \right] , \end{aligned}$$

where

15 $$\begin{aligned} \bar{\lambda }= {\mu _d^{4-n}\over 16\pi ^2}\left\{ {1\over n-4}-{1\over 2} \left[ \ln (4\pi ) +\Gamma '(1)+1\right] \right\} . \end{aligned}$$

The final expression is obtained by subtracting $B_0^{ST}$ from $B_0$. The subtracted integral $B_0^{R}$ is indeed of order $\mathcal{O}(q)$ if $p^2\sim \mu _p^2\sim m^2$, and it is of order $\mathcal{O}(Q)$ if we take $p^2\sim \mu _p^2\gg m^2$. This can easily be seen by expanding in *M* and $p^2-\mu _p^2$:

16 $$\begin{aligned} B_0^R= & {} \frac{\left( \mu _p^2-p^2\right) \left[ m^2 \ln \left( \frac{\mu _p^2}{m^2} -1\right) -i \pi m^2+\mu _p^2\right] }{\mu _p^4}\nonumber \\&-\, \frac{\left( p^2-\mu _p ^2\right) ^2 \left[ 2 i \pi m^4-2 i (\pi -i) m^2 \mu _p ^2-2 \left( m^4-m^2 \mu _p ^2\right) \ln \left( \frac{\mu _p ^2}{m^2}-1\right) +\mu _p ^4\right] }{2 \mu _p ^6 \left( m^2-\mu _p ^2\right) }\nonumber \\&- \,\frac{M^2 \left[ \left( m^2+\mu _p ^2\right) \ln \left( \frac{\mu _p ^2}{m^2}-1\right) -i \pi m^2-2 \mu _p ^2 \ln \frac{M}{m}-i \pi \mu _p ^2+\mu _p ^2\right] }{\mu _p ^2 \left( m^2-\mu _p ^2\right) }+\cdots . \end{aligned}$$

## Pion–nucleon scattering at leading one-loop order

The purpose of this section is to apply the EOMS scheme with sliding scale in the low-energy region of pion nucleon scattering at order $q^3$ and compare the results with those of the EOMS scheme. For energies in the threshold region we take the subtraction scale $\mu$ as a small quantity and therefore the standard *q* counting applies for tree as well as for loop diagrams. For larger values of $\mu$ the relative values of different contributions change, some of them becoming more important than others and the new *Q*-counting applies (note that there is no sharp border between the threshold and higher-energy regions). Applying the above specified rules of *Q*-counting, the loop diagrams of order $q^3$ contribute at order $Q^0$ and higher. Loop diagrams of order $q^4$ start contributing at order $Q^1$. That is, for higher energies our calculation of this section corresponds to the full $Q^0$ calculation of diagrams involving only pions and nucleons. For phenomenological applications in the energy region far beyond the threshold it is necessary to include relevant resonances as dynamical degrees of freedom. For example, if we are interested in $\pi N$ elastic scattering up to 1.6 GeV, in the $P_{33}$ partial wave we need to include the $\Delta (1232)$ and the $\Delta (1600)$ as explicit degrees of freedom, in the $P_{11}$ partial wave the Roper resonance *N*(1440) has to be taken into account, etc. We postpone such a comprehensive analysis for the future work.

The lowest-order standard pion–nucleon Lagrangian, generating the nucleon propagator and vertices needed in this section, is given by [3]

17 $$\begin{aligned} \mathcal{L}_{\pi N}^{(1)}= & {} \bar{\Psi }\left( i\gamma _\mu D^\mu -m + \frac{1}{2} g_A \gamma _\mu u^\mu \gamma _5 \right) \Psi , \end{aligned}$$

and the lowest-order $\mathcal{O}(q^2)$ effective mesonic Lagrangian has the form [2]

18 $$\begin{aligned} \mathcal{L}_2=\frac{F^2}{4}\text{ Tr }(\partial _\mu U \partial ^\mu U^\dagger ) +\frac{F^2 M^2}{4}\text{ Tr }(U^\dagger + U). \end{aligned}$$

**Fig. 3:**
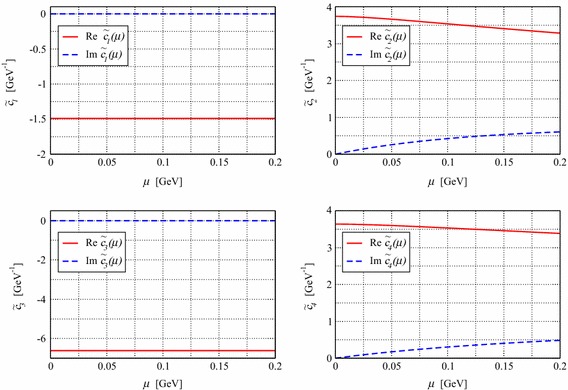
The renormalized (complex) coupling constants of EOMS scheme with sliding scale $\mu \in [0,0.2]$ GeV. The *solid* (*red*) and *dashed* (*blue*) *lines* represent the real and imaginary parts of the coupling constants, respectively

**Fig. 4:**
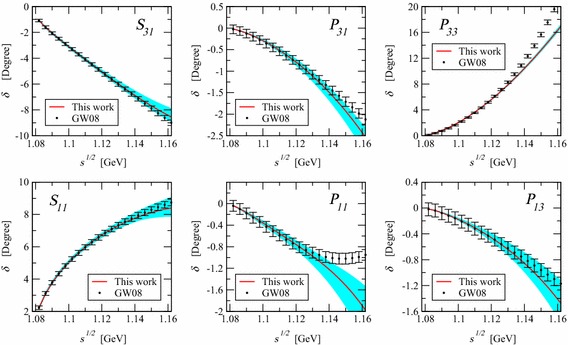
Partial-wave phase shifts. The *solid* (*red*) *lines* are our predictions using the central values of the LECs from fitting, while the *cyan bands* show the change of the phase shifts corresponding to the variation of the LECs within their $1-\sigma$ uncertainties. *Solid* (*black*) *dots* represent phase shifts taken from Ref. [26]. Note that fit has been performed for energies up to 1.13 GeV. Here the renormalization scale was taken $\mu =0$, which corresponds to the EOMS scheme

**Fig. 5:**
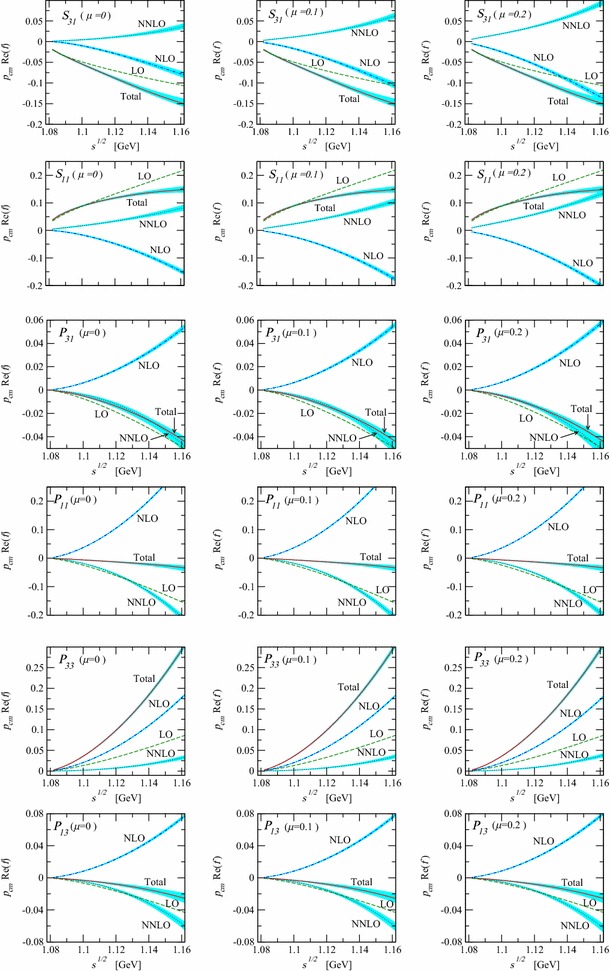
Real part of the amplitudes multiplied by the modulus of the nucleon momentum in the center of mass frame. The *dashed* (*green*), *dash*-*dotted* (*blue*), *dotted* (*violet*) and *solid* (*red*) *lines* stand for the LO, NLO, NNLO contributions and their sum, respectively. The *cyan bands* show the change of the phase shifts corresponding to the variation of the coupling constants within their $1-\sigma$ uncertainties. The corresponding values of $\mu$ are shown in the figures

The pion–nucleon Lagrangian of second and third orders, needed for our tree diagrams, can be found in Refs. [20, 21]. Tree and loop diagrams contributing to the pion–nucleon scattering at $q^3$ order are shown in Figs. 1 and 2, respectively. Contributions of tree-order contact diagrams in the pion–nucleon scattering amplitudes are given in Eq. (8). We calculated all loop diagrams and subtracted the power counting violating terms. To obtain the subtraction terms we expanded the $D^{\pm }$ amplitudes generated by the loop diagrams in powers of *M*, *t*, and $\nu ^2-\mu ^2$ up to order $q^2$ by counting *M* as order $q^1$, and *t* and $\nu ^2-\mu ^2$ as order $q^2$. As the $B^\pm$ are multiplied by , which counts as order $q^2$, we only need to subtract the zeroth-order contributions from them. We checked that all subtraction terms are absorbed by redefining the coupling constants of the effective Lagrangian. While the subtraction terms are complex for $\mu \ne 0$, we further checked that in the $\mu \rightarrow 0$ limit the real-valued subtraction terms of the EOMS scheme [24, 25] are reproduced. The complex renormalized coupling constants, i.e. $c_i~(i=1,\ldots ,4)$, of the EOMS scheme with sliding scale are shown in Fig. 3, where the subtraction scale $\mu$ varies from 0 to 0.2 GeV. We do not show the $d_i$ couplings of the third-order Lagrangian because at this order of accuracy they are all $\mu$-independent. All the involved coupling constants for $\mu =0$ case are determined, following the strategies of Refs. [24, 25], by fitting to the phase shifts of the GWU/SAID group [26] and results very similar to those of Refs. [24, 25] are obtained, they are shown in Fig. 4. For $\mu \ne 0$, the coupling constants can be obtained with the help of renormalization group equations with respect to $\mu$, which lead to

19 $$\begin{aligned} \tilde{c_i}(\mu )= c_i^\mathrm{EOMS}+\frac{m}{32\pi ^2F^2}\delta _i(\mu ),\quad c_i^\mathrm{EOMS}\equiv \tilde{c_i}(\mu =0),\nonumber \\ \end{aligned}$$

where the explicit expressions of $\delta _i(\mu )$ are given in the appendix. The real parts of the amplitudes for three specific subtraction scales, $\mu =0$, 0.1, and 0.2 GeV, are shown in Fig. 5. As expected from general considerations, the relative size of contributions of different orders depends on the choice of $\mu$.

The bare parameters expressed in terms of renormalized ones are substituted in the effective Lagrangian generating the main interaction terms and counterterms. These counterterms need to be fixed once, in our case by adjusting them to subtraction terms of the pion–nucleon scattering amplitudes. For other processes, where the same terms of the effective Lagrangian contribute, the same renormalized couplings and counterterms are used. This guarantees that our approach respects all underlying symmetries encoded in the effective Lagrangian. While we cannot give a general proof that the same counterterms also remove the power counting violating terms from loop diagrams contributing to various related processes, we expect that this is the case. The reason for this is that the Ward identities derived from symmetries of the effective Lagrangian are satisfied order-by-order of the expansion around any kinematical point, not only at threshold.

## Conclusions

In this work we introduced a new approach to BChPT which is applicable for processes in the one-nucleon sector at small scattering angles at energies beyond the low-energy threshold regions. In this kinematical region, despite the higher energies, the quark structure of hadrons is still not resolved. For the considered energies contributions of tree-order diagrams have to be re-ordered. This is done by re-arranging the chirally invariant terms of the standard low-energy effective Lagrangian. Resonances which appear for the considered energies need to be included as explicit degrees of freedom. This guarantees that the tree-order diagrams represent Taylor series expansions of analytic functions and thus are convergent. This way we obtain an effective field theoretical approach with a well-defined power counting for tree diagrams. To apply the same power counting also for loop diagrams, we use a new renormalization scheme for loop diagrams, the generalized EOMS scheme with sliding scale. Within this scheme, by exploiting the freedom of the choice of renormalization condition in quantum field theories, we shift the renormalization point in the physical region beyond the threshold. The renormalized loop diagrams satisfy a systematic power counting for higher energies, provided that small scattering angles are considered. By shifting the renormalization point in the physical region beyond the threshold we subtract also the imaginary parts of loop diagrams. This requires splitting of real bare couplings in complex renormalized couplings and complex counterterms. Thus the renormalized coupling constants of our re-arranged effective Lagrangian become complex. Finally, we are left with a self-consistent effective field theoretical approach with a well-defined power counting. The new re-arranged effective Lagrangian contains a finite number of terms at any finite order and a finite number of Feynman diagrams contribute to physical quantities at any finite order. In the current work we have not included resonances, but rather concentrated on conceptual issues of the pion–nucleon sector. While we considered only the pion–nucleon scattering here, the pion photo- and electro-production processes as well as Compton scattering and processes involving several pions and/or photons (for special kinematics) can be treated analogously.

## Appendix A: Running of the dimension-two LECs

Explicit expressions of the coefficients $\delta _i(\mu )$ appearing in Eq. (19) are given by

A1 $$\begin{aligned} \delta _1(\mu )&=0,\nonumber \\ \delta _2(\mu )&=\bigg \{-\frac{(1+g_A^2)^2}{(m^2-4\mu ^2)}\bar{\mathcal {H}}_{01}+\frac{g_A^4m^2}{\mu ^2}\bar{\mathcal {H}}_{11}(m^2)\nonumber \\&\quad -\bigg [\frac{\bar{\mathcal {H}}_{11}(m^2+2m\mu )}{2\mu ^2(m+2\mu )}\bigg (4g_A^2\mu ^3-2\mu ^2(m+\mu )\nonumber \\&\quad +g_A^4(m^2+3m^2\mu -2\mu ^3)\bigg )\nonumber \\&\quad +\frac{g_A^4m^3}{\mu }\bar{\mathcal {H}}_B(m^2+2m\mu )+(\mu \rightarrow -\mu )\bigg ]\bigg \}_{M^2=0}\nonumber \\&\quad -\left\{ (g_A^2-1)^2\log \left( \frac{m^2}{\mu _d^2}\right) -(2+g_A^4)\right\} \,\nonumber \\ \delta _3(\mu )&=0 \nonumber \\ \delta _4(\mu )&=\bigg \{-\frac{g_A^2(-5+g_A^2)}{4}\nonumber \\&\quad +\frac{-(2+7g_A^2+3g_A^4)m^2+4(1+5g_A^2+2g_A^4)\mu ^2}{4m^2(m^2-4\mu ^2)}\bar{\mathcal {H}}_{01}\nonumber \\&\quad +\frac{g_A^2(-1+5g_A^2)}{4}\bar{\mathcal {H}}_{02}(0)+g_A^2\left( 1+\frac{2g_A^2m^2}{\mu ^2}\right) \bar{\mathcal {H}}_{11}(m^2)\nonumber \\&\quad +\bigg [\frac{\bar{\mathcal {H}}_{11}(m^2+2m\mu )}{2m\mu ^2(m+2\mu )}\bigg (\mu ^4-2g_A^2\mu ^3(m+\mu )+g_A^4 \nonumber \\&\quad \times (-2m^4-8m^3\mu -7m^2\mu ^2+2m\mu ^3+\mu ^4)\bigg )\nonumber \\&\quad -\frac{g_A^4m^2(m+\mu )}{\mu }\bar{\mathcal {H}}_B(m^2+2m\mu )+2g_A^4m^4\bar{\mathcal {H}}_{13} \nonumber \\&\quad \times (m^2+2m\mu ,0)+(\mu \rightarrow -\mu ) \bigg ]\bigg \}_{M^2=0}\nonumber \\&\quad -\frac{1}{2}\left\{ (3g_A^4-2g_A^2-1)\log \left( \frac{m^2}{\mu _d^2}\right) -g_A^2(5+g_A^2)\right\} . \end{aligned}$$

Here, the loop integrals are defined as

A2 $$\begin{aligned} \begin{aligned}&\mathcal {H}_{01}=\frac{(2\pi \mu _d)^{4-n}}{i\pi ^2}\int {\mathrm{d}^nk}\frac{1}{k^2-m^2}\ ,\\&\mathcal {H}_{11}(s)=\frac{(2\pi \mu _d)^{4-n}}{i\pi ^2}\int {\mathrm{d}^nk}\frac{1}{[k^2-M^2][(k-p-q)^2-m^2]}\ ,\\&\mathcal {H}_{02}(t)=\frac{(2\pi \mu _d)^{4-n}}{i\pi ^2}\int {\mathrm{d}^nk}\frac{1}{[(k-p)^2-m^2][(k-p^\prime )^2-m^2]}\ ,\\&\mathcal {H}_{B}(s)=\frac{(2\pi \mu _d)^{4-n}}{i\pi ^2}\int {\mathrm{d}^nk}\frac{1}{[k^2-M^2][(k-p)^2-m^2][(k-p-q)^2-m^2]}\ ,\\&\mathcal {H}_{13}(s,t)=\frac{(2\pi \mu _d)^{4-n}}{i\pi ^2}\int {\mathrm{d}^nk}\frac{1}{[k^2-M^2][(k-p)^2-m^2][(k-p-q)^2-m^2][(k-p^\prime )^2-m^2]}\ . \end{aligned} \end{aligned}$$

Note that *p* ($p^\prime$) and *q* are the momenta of the incoming (outgoing) nucleon and of the incoming pion, respectively. The finite parts of the loop integrals that remain after subtracting the UV divergent parts proportional to $\bar{\lambda }= {\mu _d^{4-n}\over 16\pi ^2}\left\{ {1\over n-4}-{1\over 2} \left[ \ln (4\pi ) +\Gamma '(1)+1\right] \right\}$ are denoted as $\bar{\mathcal {H}}$.

## Appendix B: Complex renormalized parameters and unitarity

The use of complex renormalized parameters raises the question if the CMS violates unitarity. In general, unitarity is guaranteed by a real (Hermitian) bare Lagrangian and the fact that the renormalization is an identical transformation. Still, order-by-order unitarity in perturbation theory within the CMS is a non-trivial issue. It has been looked at in Ref. [19] and thoroughly investigated recently in Ref. [18]. Here, we give an intuitive argument for demonstration. For definiteness, let us consider the scalar $\phi ^4$ theory in 4 dimensions. The Lagrangian of the theory depends on two parameters, the bare mass $m_0$ and the bare coupling $\lambda _0$. Using standard dimensional regularization and the minimal subtraction scheme (MS) we get rid of the divergences and express the physical quantities, like the scattering amplitudes, in terms of renormalized parameters of the MS scheme $m_{MS}$ and $\lambda _{MS}$ as power series in the renormalized coupling constant

B1 $$\begin{aligned} M_i= F_i(m_{MS}(\mu _d),\lambda _{MS}(\mu _d), p,\mu _d), \end{aligned}$$

where *p* stands for kinematical variables and $\mu _d$ is the renormalization scale. The physical amplitudes satisfy the conditions of unitarity up to the order of accuracy of a given calculation. We can change the renormalization scheme by switching to another one. For example, we can calculate the pole mass of the scalar particle and the scattering amplitude *M*(*s*, *t*, *u*) of two scalars at the symmetric non-physical kinematical point

B2 $$\begin{aligned}&m = \phi _1 (m_{MS}(\mu _d),\lambda _{MS}(\mu _d),\mu _d),\nonumber \\&\lambda (\bar{\nu })= M(-\bar{\nu }^2/3,-\bar{\nu }^2/3,-\bar{\nu }^2/3)\nonumber \\&\quad = \phi _2 (m_{MS}(\mu _d),\lambda _{MS}(\mu _d),\bar{\nu },\mu _d), \end{aligned}$$

express $m_{MS}(\mu _d)$ and $\lambda _{MS}(\mu _d)$ in terms of *m* and $\lambda (\bar{\nu })$ from Eq. (B2) and substitute in Eq. (B1). This way we obtain

B3 $$\begin{aligned} M_i= \tilde{F}_i(m,\lambda (\bar{\nu }), p,\bar{\nu }), \end{aligned}$$

where the $\tilde{F}_i$ are some functions (different from $F_i$) of real arguments. Surely enough by doing this identical transformation one does not violate unitarity.

Although very convenient, it is by no means necessary to choose the new renormalized coupling at a non-physical kinematical point. Taking e.g. a physical normalization point

B4 $$\begin{aligned}&m = \phi _1 (m_{MS}(\mu _d),\lambda _{MS}(\mu _d),\mu _d),\nonumber \\&\lambda _C(\bar{\nu }) = M(2 m^2+\bar{\nu }^2,0,2 m^2-\bar{\nu }^2)\nonumber \\&\qquad = \phi _3 (m_{MS}(\mu _d),\lambda _{MS}(\mu _d),\bar{\nu },\mu _d), \end{aligned}$$

expressing $m_{MS}(\mu _d)$ and $\lambda _{MS}(\mu _d)$ in terms of *m* and $\lambda _C(\bar{\nu })$ from Eq. (B4) and substituting in Eq. (B1), we obtain

B5 $$\begin{aligned} M_i= \bar{F}_i(m,\lambda _C(\bar{\nu }), p,\bar{\nu }), \end{aligned}$$

with $\bar{F}_i$ some functions (different from $\tilde{F}_i$ and $F_i$) of real and complex arguments. Once more, by doing this identical transformation one does not violate unitarity, even though $\lambda _C(\bar{\nu })$ is complex. However, as the unitarity condition is only satisfied up to higher orders of perturbation theory, the relevant issue is of course the convergence of the obtained perturbative series.
